# An Animal Welfare Risk Assessment Process for Zoos

**DOI:** 10.3390/ani8080130

**Published:** 2018-07-28

**Authors:** Sally L. Sherwen, Lauren M. Hemsworth, Ngaio J. Beausoleil, Amanda Embury, David J. Mellor

**Affiliations:** 1 Department of Wildlife Conservation and Science, Zoos Victoria, Victoria 3052, Australia; aembury@zoo.org.au; 2 The Animal Welfare Science Centre, the University of Melbourne, Victoria 3051, Australia; lauren.hemsworth@unimelb.edu.au; 3 Animal Welfare Science and Bioethics Centre, School of Veterinary Science, Massey University, Palmerston North 4474, New Zealand; N.J.Beausoleil@massey.ac.nz (N.J.B.); D.J.Mellor@massey.ac.nz (D.J.M.)

**Keywords:** zoo, welfare, assessment, Five Domains

## Abstract

**Simple Summary:**

To retain social license to operate, achieving and maintaining high standards of animal welfare need to be institutional priorities for zoos. In order to be confident in the delivery of high standards of animal welfare, a holistic evidence-based approach to welfare assessment is required. This should include a combination of institutional-level assessments, individual animal monitoring tools, and applied research targeted at advancing our understanding of species needs and preferences in zoos. Progress has certainly been made in the zoo sector in development of research programs and individual animal welfare monitoring tools. Comparatively less focus has been applied to institutional-level assessment processes. This paper aims to fill this gap and presents an outline of a welfare risk assessment process developed and trialed at three zoos over a three year period and discussion of the potential value it offers, as well as the limitations of its use.

**Abstract:**

There is a growing interest and need for zoos to develop and implement welfare assessment tools that are practical to use and provide meaningful results that can inform management decisions. This paper presents a process that was developed to support this type of evidence-based management in zoo animal welfare. The process is configured to facilitate institutional risk assessment, using an adapted version of the Five Domains Model for animal welfare assessment. It is designed to systematically analyse information gathered from zoo personnel in order to highlight areas of welfare risk, as well as areas that are performing well and areas requiring further investigation. A trial was conducted on three zoos over three years. Results of the trial suggest the process developed is practical and effective in identifying areas of welfare risk in a wide range of species in a zoo setting. It represents a further step towards achieving high-level animal welfare in zoos by integrating animal welfare as an institutional priority. The more zoos that employ such strategies, the greater the ability of the sector to advance the welfare of the animals in their care.

## 1. Introduction

Over the past decade, members of the public have shown increasing interest in the welfare of animals in human care [1,2,3,4]. It has been suggested that increased scientific understanding of the sentience of some animals combined with assessments of potential impacts on their welfare have driven ethical reflection and ultimately sectorial change [5]. Such scrutiny may be expected to intensify as our scientific understanding of animal welfare develops further [6,7], especially as social media will continue to facilitate rapid and widespread dissemination of this information, enabling animal welfare discussions to become more mainstream.

Retention of social license to operate under this intensifying enquiry will require zoos and aquariums to have robust ethical foundations [8]. Without clear ethical principles to guide them, zoos and aquariums risk taking actions that will be challenged, undermining the core of their operations. Reflecting these ethical foundations, the sector needs to demonstrate carefully considered justification for housing living animals, a solid commitment to superior animal welfare standards, and an empirical operating philosophy that fosters continuous improvement. For many modern zoos, the justification for housing animals in captivity is for the benefit of conservation [9,10,11]. However, it is clear that even the most ambitious conservation goals will not be adequate justification if a zoo does not actively demonstrate high standards of animal welfare.

Research demonstrates that people care about animal welfare standards in wildlife tourist attractions and, when primed, exhibit a preference for attractions that showcase conservation value and good animal welfare [12]. The general public also appear able to identify some forms of abnormal behaviour in captive animals. For example, after watching a short video of either a tiger pacing or resting, peoples’ survey responses indicated that viewing a tiger pacing significantly decreased both their perception of the level of care animals received and their interest in supporting zoos [13]. Accordingly, any signs of suffering or welfare compromise in zoo-housed animals can impede a zoo’s contribution to conservation due to the reliance on visitation to support conservation outcomes financially and as the targets of education campaigns. Thus, both achieving and maintaining high standards of animal welfare need to be institutional priorities for zoos, underpinning all their conservation work.

### 1.1. Need for Appropriate Welfare Strategies in Zoos

Having appropriate animal welfare strategies can help zoos to be progressive and proactive in their achieving high welfare standards. These strategies need to demonstrate commitment to applied welfare research, individual welfare monitoring and regular assessments of potential risks to, and opportunities to enhance, animal welfare. Indeed, the Zoo and Aquarium Association in the Australasian region has an animal welfare-based accreditation process that requires institutions to have documented guiding principles with respect to animal welfare. Applied research should be used to inform these policy documents. There is a growing body of published work on applied animal welfare science in the zoo sector which explores the welfare consequences of various factors such as enrichment provision [14,15,16], enclosure design [17,18,19], social groupings [20], and human impacts [21,22,23,24]. This type of research is valuable in informing housing and husbandry standards for zoos, and clearly further study is needed to address the wide taxonomic spread of species housed in these settings. However, these studies can be resource intensive, often take a long time and are usually focused on a specific research question. Whilst there is clearly a parallel need for more holistic welfare assessments to complement information gathered from these focused empirical studies, to date, comparatively little research has been conducted on welfare assessment techniques in zoos. Such assessments may have a resource-based or animal-based focus.

### 1.2. Resource-Based and Animal-Based Welfare Assessments

Monitoring the provision of welfare-relevant resources such as housing, food, and water is based on the assumption that if the appropriate resources are provided, the animals’ welfare will be good [25,26]. This approach is an indirect means of monitoring animal welfare which is advantageously based on objective measures [25], thereby making assessments easy to conduct in situ. However, resource provision does not necessarily translate to good welfare [25,27,28,29] even if strict monitoring procedures are in place.

In contrast, animal-based welfare monitoring, recognised for more than a decade in the farm animal sector [25,30,31,32,33], is based on indices of physical condition, physiological state, and behaviour. This approach accommodates significant variation in how individual animals respond when provided with the same resources, variability being attributable to a range of factors including temperament and interactions between genetic makeup and the environment [26,32,33]. Its advantage is the direct insights it gives about the welfare of individual animals [34]. However, both resource-based and animal-based approaches are recognised as providing useful welfare-relevant information [30,35].

### 1.3. Zoo Animal Welfare Assessment Approaches

To date, most welfare monitoring attempts in zoos have focused on the development of individual animal welfare monitoring tools. Welfaretrak is a tool based on caretaker assessments of an individual’s welfare over time by completing brief weekly surveys [28]. The surveys include both physical and emotional welfare indicators and the website tracks responses over time, highlighting any changes in individual welfare score. It is based on the rationale that zoo keepers can serve as proxy informants for the animals they work with as they have the most familiarity with the animal’s temperament and preferences, and can detect even subtle changes in their behaviour [29]. Furthermore, the inter-observer agreement of ratings performed by zookeepers or experienced observers on zoo animals has been examined in a number of studies, and overall, high levels of agreement have been reported [29,36,37,38]. For example, Clouded Leopards in North American zoos that were highly rated by their keepers as “tense” and “pacing” were found to have higher overall faecal glucocorticoid metabolite concentrations than leopards whose keepers did not rate them highly for these traits [38].

Researchers at Disney’s Animal Kingdom have also designed an animal welfare monitoring program for individual animals that tracks animal-based measures including behavioural and endocrine data, keeper scores on parameters such as fur condition, diet consumption, exploration, and sleeping behaviour; they also track resource-based measures including husbandry events and environmental measures such as noise levels [39].

An adaptation of the Animal Welfare Assessment Grid (AWAG) has been applied to a selection of zoo primates and birds [40]. The AWAG is a computer-based system, originally designed for research institutions. It focuses on four parameter classes designated “physical”, “psychological”, “environmental”, and “procedural”. Within each class, users are asked to give a numerical score from 1–10 for each individual or group and the results are plotted on a grid to allow easy visualisation of relative parameter scores. 

Lastly, there is the Cetacean Welfare Assessment process (C-Well) that has been developed for use on individual monitoring of captive bottlenose dolphins [41]. This tool is based on the European Welfare Quality^®^ animal welfare assessment protocol for farm animals which focuses on scoring animal-based indicators associated with four main principles deemed necessary for good animal welfare; good feeding, good housing, good health, and appropriate behaviour [42].

Focusing more on the institutional level, Kagan et al. [43] proposed a universal animal welfare framework for zoos that included two separate survey tools for welfare evaluation. One focused on examining zoo practices, policies, and resources and the other focused on individual animals in relation to their housing, routine, and behaviour. The tools were designed to be used together to collect qualitative information to reveal the current state of welfare processes and programs.

There are also programs such as Zoo Monitor [44] and AWARE [45] that offer technological support in welfare monitoring for zoos through the use of specially designed software programs to facilitate data collection. 

### 1.4. Developing an Animal Welfare Risk Assessment Process

The above assessment processes largely focus on individual animal welfare monitoring protocols (excluding aspects of Kagan et al., [43]) that involve frequent assessments to identify and flag welfare problems as they arise. As such, they play an important role in safeguarding zoo animal welfare. However, the zoo sector remains limited in its ability to conduct proactive, institutional-level assessments of welfare risk factors across multiple species that can inform prioritisation of resources and be used for benchmarking progress in advancing welfare standards more broadly. 

The Five Domains Model provides a useful, practical framework to facilitate this form of welfare risk assessment [46,47,48]. The Model was originally configured to provide a systematic, coherent, and inclusive method for identifying welfare compromise by reference to four physical domains (health, nutrition, environment, and behaviour) and one mental domain (to reflect the animal’s overall affective experience). However, more recently, the Model was updated to also facilitate identification of rewarding experiences that may be associated with positive affective states [48].

Central to effective application of a framework aiming to facilitate the progressive improvement of zoo-based welfare standards is the higher standard against which current welfare findings are to be compared. The usual approach to driving improvements has been to first meet, and then progressively increase minimum standards where possible. However, simply focusing on this usual approach is likely to result in smaller changes than if current standards were compared with higher standards based on offering more opportunities for animals to have rewarding experiences [47]. In the zoo setting this is complicated by the diversity of species housed and limits to welfare-relevant knowledge of many of the species. However, a good starting point may be to consider species-specific natural behavioural biology when making judgments about the acceptability or otherwise of current conditions and when devising strategies to improve them [31,47,49]. It is apparent that, even for relatively unexpressive animals such as snakes, the closer an animal’s surroundings and social circumstances are to its natural living conditions, the more it will be able to express species-specific behaviours and the better its welfare may be.

This thinking has been incorporated into the development of a novel welfare risk assessment process for zoos. This paper presents an outline of the process, a trial of its use at three zoos over a three-year period and discussion of the potential value it offers, as well as the limitations of its use. The process is configured to facilitate institutional risk assessment, using an adapted version of the Five Domains Model for animal welfare assessment. It is designed to systematically analyse information gathered from zoo personnel in order to highlight areas of welfare risk, as well as areas that are performing well, and areas requiring further investigation.

## 2. Materials and Methods—Description of Process

### 2.1. Sites

This process in its current form was tested for three consecutive years (2015–2017) at three zoos in Australia. A total of 628 assessments were conducted in this period across all sites including a range of mammals, birds, fish, reptiles, amphibians, and invertebrates (with over 339 species). Assessments were conducted on all enclosures that were housing animals at each zoo.

### 2.2. Description of the Process

The process was designed to systematically collect information from a team of experienced zoo personnel who included zoo keepers, veterinarians, managers, and a welfare researcher/specialist to allow potential and/or current risks to animal welfare to be identified. The process was intended for annual use at each site. 

The process consists of a total of 20 indicators, including 15 resource-based welfare risk factors and 5 animal-based measures (Table 1). These indicators/measures were categorised across an adapted version of the four physical/functional domains of the Five Domains Model [35], reconfigured as (1) “Environment (physical/social)”; (2) “Behaviour”; (3) “Physical Health/Nutrition”; and (4) “Husbandry”. Husbandry was included as the last domain to recognise the potentially profound impact human presence, e.g., as keepers and/or visitors, can have on zoo animals [23,24,50].

For each zoo department, the team of personnel completed the assessment together for each of their species/enclosures. Group judgements were made on each indicator by scoring as described in Table 2, using the team’s best understanding of the species’ natural behavioural biology as the gold standard benchmark. These numerical scores were designed to facilitate analysis of what is really a qualitative assessment.

Assessments were conducted on the enclosure level. For group housed animals, the animal-based measures were assigned based on the lowest scoring animal. For example, if one individual in the group frequently displayed abnormal behaviour, the enclosure would score a 0 for this measure.

During the assessment, the team discussed each indicator/measure until agreement was reached on the score. If agreement could not be reached, the animal welfare specialist would make the final judgement. Although data collection occurred annually at the same time of year (between October–December each year), judgements were based on conditions animals experienced year-round, inclusive of all seasons. 

Assessments were completed directly into a web-based survey form on the staff intranet at the organisation. There were four pages to complete, one for each welfare domain, with a space at the bottom of each page to enter any comments. On average an assessment took 35 min for an enclosure (however this varied according to the species and discussion within the group, with some taking up to 50 min for one enclosure). Data were then exported directly to Microsoft excel for analysis.

### 2.3. Data Analysis

For each enclosure an average overall welfare risk score was calculated by averaging the scores of all 20 indicators/measures. This score can range from 0 (considered the highest overall welfare risk) to 2 (the lowest overall welfare risk, representing most positive overall welfare score). The dataset of these average welfare risk scores for each zoo in each year is then analysed to provide descriptive statistics including mean, median and variance.

From the full dataset of 220 enclosures across the three zoos in 2015, the spread of average welfare risk scores was analysed to determine a threshold for the lowest 5th percentile of enclosures. The enclosures falling below this threshold were categorised as ‘at highest risk’ and recommended for urgent welfare intervention. This threshold was determined to be a score of 1.19 or lower. This was maintained as the set threshold for all future years of assessment to try to create a benchmark for progress, despite the spread of data changing in the following two years. The score of 1.19 out of 2 still represents a score of almost 60% of the possible maximum which may not appear to be considered high risk compared to other forms of risk assessment, however the team decided at the outset to benchmark at a relatively high standard. The distribution of each dataset of average welfare risk scores was negatively skewed (i.e., many high scores and fewer low scores), thus medians were used to investigate institutional changes across years and any differences across taxa.

In addition to evaluating data on the enclosure level, data were also analysed on the indicator/measure level within zoos. Average welfare risk scores for indicators/measures were calculated by averaging all enclosure scores for that indicator/measure. Median and mean scores for each welfare domain were calculated to evaluate levels of performance in each area. The two lowest scoring indicators/measures were considered the poorest performing that year and the two highest were considered the best performing. Benchmark scores were not set for these as highlighting them was intended to raise general awareness around the indicator/measure to encourage general intention of developing welfare strategies for each zoo.

Lastly, the total number of indicators/measures scored as ‘unknown’ was tallied for each enclosure and for each indicator/measure to identify the organisations gaps in knowledge and a need for further investigation.

### 2.4. Reporting Results

The aim was to use the results of the welfare risk assessment to prioritise interventions for enclosures, as well as to introduce remedial actions across zoos in the poorest scoring areas. To facilitate this, a report was developed using each year’s collated data and presented to teams at each zoo. The purpose was to summarise the results and make recommendations for the welfare work plan for the following 12 months with the aim of addressing and reducing welfare risks and improving the assessment scores.

The highest priority actions were highlighted for the enclosures falling below the 1.19 score threshold as these were deemed to represent the highest risk for each zoo. Furthermore, the enclosures and indicators/measures identified to have the most ‘unknown’ scores were recommended as targets for research priority.

In addition, each department manager received the entire dataset and was able to develop their own plan to address each of the indicators/measures scored as 0 in their department. Progress in terms of intervention delivery (e.g., installation, renovation, and shift in regime) was tracked and reported to the senior executive team monthly. 

## 3. Results 

### 3.1. Demonstration of Use

A total of 628 assessments, involving over 339 species across a range of taxa were conducted at the three zoos over a three-year period. The number of enclosures assessed each year at each zoo is outlined in Table 3. This represented over 95% of all enclosures housing animals at each zoo, each year. 

**Figure 1:**
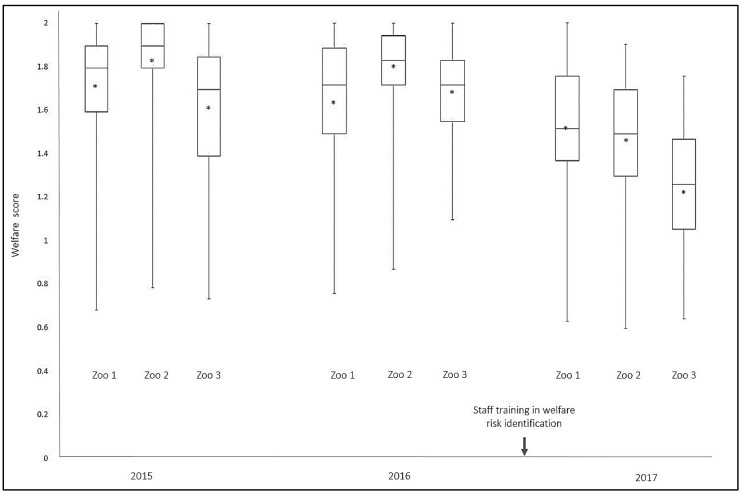
Box plot showing median welfare risk, upper and lower quartiles, the minimum-to-maximum range and mean welfare risk scores (*) at each zoo over the 3-year trial. The higher the welfare score, the lower the risk identified.

Overall, welfare scores were relatively high (signifying low welfare risk), especially in the first two years of data collection (Table 3). All three zoos showed similar trends in variation in welfare scores over the three years (Figure 1), with the most recent assessments in 2017 showing the lowest scores for that period. The lower average welfare scores in 2017 were associated with an increase in the total number of risks identified in that year. 

### 3.2. Indicator Level Evaluation

The three risk factors with the lowest scores (higher welfare risk) throughout the 3-year trial were sensory environment, climate range and observation time for keepers. The highest scoring (low welfare risk) indicators/measures were body condition, provision of proactive health care, and good relationship with keepers, and finally, the indicators/measures that were scored as ‘most unknown’ were desirable time budgets, abnormal behaviour, and behavioural diversity.

### 3.3. Taxonomic and Welfare Domain Trends

**Figure 2:**
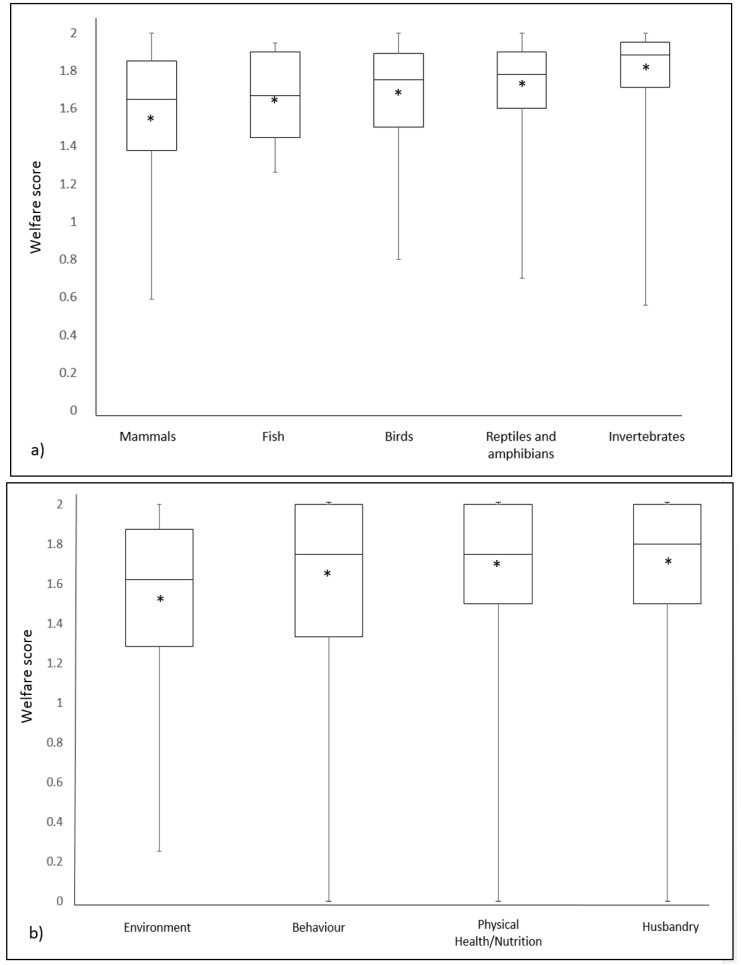
Box plots showing median welfare risk scores, upper, and lower quartiles, the maximum and minimum range and mean welfare risk scores (*) across all three zoos by taxon (**a**) and welfare domain (**b**). The higher the welfare score, the lower the risk identified.

On average across all three zoos over three years, welfare risks scores were relatively similar across taxa and domains (Figure 2a,b). Mammals received the lowest mean and median welfare score and invertebrates the highest (Figure 2a). Physical health/nutrition was the highest scoring domain of welfare and the Environment was the lowest scoring domain (Figure 2b).

## 4. Discussion

### 4.1. Interpretation of Trial Results

The trial of the welfare risk assessment process demonstrated its practicality and effectiveness in identifying areas of welfare risk in a wide range of species in a zoo setting. The decrease in average welfare scores at each of the three zoos in 2017 reflects an increase in the number of welfare risks identified. At first glance, this may appear to suggest a deterioration in conditions and welfare level over time. However, upon further consideration, many of the welfare risks newly identified in the 2017 assessments were in fact present in the two previous years but were not identified or scored to the same degree. There appear to be three possible explanations for this outcome: the conditions themselves did in fact deteriorate, the basis for judgement by the staff changed, or both. 

Staff experience, education, and awareness of animal welfare are obviously key elements in effective utilization of these processes. It is noteworthy, therefore, that one month prior to data collection for the 2017 round, a three-day staff training workshop was held for keepers and veterinarians across the three zoos. The workshop covered topics on factors that are known to influence welfare in captive animals, scientific assessment of animal welfare and tools to accurately identify and articulate risks to welfare. The majority of staff who completed the assessments were in attendance at this workshop, so it is possible that this training and open communication around animal welfare increased their awareness of, and ability to, identify and report welfare risks leading to lower welfare scores in 2017. Unfortunately, no competency-based assessments were conducted on staff before and after their workshop attendance, so the effect of this form of education on zoo personnel competency and thus the scoring outcomes remains unknown. This is something that is worth investigating in the future as assessing the inter- and intra-observer reliability of the assessment team would both aid in validating the welfare risk assessment process, and evaluating the effectiveness of education or training delivered by the organisation. Nevertheless, any improvement in staff ability to identify and report welfare risks and develop strategies to advance welfare standards proactively is an important factor in driving positive change. As such, this aspect of the results should be considered to be positive from a motivational perspective. Furthermore, as staff become more familiar with the assessment process and gain experience identifying welfare risks, it is expected that their ability to make judgements around welfare risks will be enhanced.

It is also possible that in the first two years of data collection there was a bias towards more positive scoring because of the perception that welfare standards reflect the quality of keepers’ care for their animals. For this reason, it is important that the use of such processes is premised with clear communication around the primary role of the process being to identify opportunities for animal welfare improvements, not to detect deficiencies in staff delivery of care.

Considering the highest and lowest scoring indicators/measures, it is interesting that animal observation time was scored by staff as a common risk factor in their ability to deliver high standards of welfare. This is logical as keepers are expected to be the ‘voice’ for the animals in their care. They have the best understanding of the individual animal’s temperament and preferences and can detect subtle changes in their behaviour [29]. It is likely that this ability to detect changes in observable welfare indicators in individuals is heavily influenced by the time that keepers spend observing the animals, and ultimately their familiarity with their behaviour. Alternatively, it is possible that keepers gain a somewhat distorted picture of the animal’s behaviour if the animals they work with recognise them as sources of food, enrichment, and/or other positive or negative events. For this reason, it is beneficial to facilitate processes that allow keepers to conduct systematic behavioural observations in ways that do not interfere with the animals’ behaviour. This was the rationale for the installation of various new CCTV networks in enclosures in 2017, based on the welfare priorities identified through the use of this assessment process. Remote monitoring of animals can also allow noninvasive assessment of animals for those at risk of adverse responses to disturbance (e.g., nocturnal species, shy species, and animals in recovery from veterinary procedures).

The sensory environment was also identified as a significant welfare risk factor, suggesting that the keepers believed this to be an environmental feature that posed certain risks to animals. This is in line with the recent increase in published studies investigating the impacts of various sensory conditions on animals in zoo environments such as noise exposure [39,51,52], light conditions [53], and visual stimuli [24,54]. There also appears to be increasing awareness that we do not yet have a good understanding of the breadth of sensory perception across the multitude of species housed in zoos, and as a result the impact of the zoo environment on these animals [39].

In terms of high-scoring indicators, keeper relationships with animals were scored positively. It is well recognised in other settings that animal caretakers can have profound impacts on welfare [50], but few studies have examined keeper impacts on animal welfare in the zoo industry. In an investigation into reproductive success in a variety of small felid species in zoos, a positive relationship was observed between reproductive success and a husbandry style where keepers spent a considerable amount of time talking to and interacting with the cats, referring to this keeping style as high-quality [55]. A study of 72 Clouded Leopards across 12 zoos in the USA revealed that lower faecal glucocorticoid concentrations were associated with keepers spending a greater amount of time with the leopards and fewer keepers caring for the animals [38]. In both studies the authors linked these husbandry factors to the keepers’ ability to form high quality relationships with their animals. Moreover, many zoo professionals report that they have established bonds with the animals in their care, particularly with primates and carnivores [56]. It follows that the identification of a high-quality relationship between keepers and animals in this assessment process is a promising trend. 

The assessments also highlighted that the main gaps in knowledge existed in the animal behaviour domain, including measures such as behavioural diversity, frequency of abnormal behaviour, and activity budgets. This is likely associated with the low scores for ‘animal observation time’ for keepers. If keepers feel they do not have enough time to observe the animals, they are also likely to feel that they do not have a solid understanding of how the animals spend their time and what behaviours they engage in. Furthermore, some species housed in these zoos are very rare and not well studied in the wild, creating a lack of understanding of behavioural biology for the species, making it very difficult to judge what is considered ‘normal’ behaviour. This is an important gap for zoos to address by investing in both in situ and ex situ research programs. 

With regards to taxonomic differences, mammals received the lowest average welfare score compared to birds, fish, invertebrates, and reptiles and amphibians. This may be because there is a greater understanding of factors that can affect the welfare of mammals and therefore a greater capacity to identify risks. Comparatively, there are fewer studies on reptile, bird, and fish welfare in zoos [57]. Likewise, the lowest scores assigned in the Environment domain across taxa may be due to an enhanced ability to identify risks in this domain. The main environmental features do not change frequently in zoos, so environmental assessments can be made readily and frequently without a need for ongoing observation as is required for animal behaviour assessments. 

### 4.2. The Value of the Process

Safeguarding animal welfare and continuous improvement in housing and husbandry of the animals living in zoos is arguably the biggest opportunity the zoo sector faces with so many exotic species having divergent species-specific and individual requirements. In order to be confident that welfare standards are improving, an evidence-based management approach should be encouraged in zoos [58], including advanced understanding of diverse species requirements, as well as individual animal preferences and motivations. Research collaborations with Universities or other scientific organizations may prove especially useful in developing this knowledge base. Studies for acquiring the evidence for such approaches can range in scale from more detailed and narrowly focused experiments to broader assessments of welfare risks. It is apparent that a range of tools are needed to assist the zoo sector to understand, assess, and ultimately advance welfare standards. Kagan et al., [43] proposed an animal welfare framework for zoos that included two tools designed to collect qualitative information about an institution’s processes and programs, with some similar focus areas to the present risk assessment process (e.g., evaluation of climatic conditions, social groups, and enrichment provision). The present process builds upon the Kagan et al. [43] framework, in the context of the Five Domains Model, and uses numerical scores to facilitate analysis of a qualitative assessment.

The present welfare risk assessment process has demonstrated value by focusing specifically on risk assessments that can be conducted annually to help zoos prioritise areas for action and benchmark progress. Where resources are limited (funding, time, staff, etc.), it is important to be able to appropriately allocate resources in ways that will achieve the most productive results. To facilitate this, systematic processes that collate and analyse relevant information are important. As a result of this type of prioritisation, the three zoos involved in the present trial were able to develop effective welfare work plans and action a substantial number of welfare interventions as well as prioritise development of plans for large-scale enclosure renovations, in a process that places greatest priority on animal needs during decision-making. Furthermore, the ability to track progress over time is also of value. Benchmarking enables institutional welfare goals to be set and then targeted, which is an effective means of integrating animal welfare into the organisation and fostering a culture of continuous improvement. However, it is important that aspirational but realistic targets are set to achieve this outcome.

Specifically, as a result of this trial and the systematic analysis of welfare risks across the three zoos, efforts directed to advance welfare have been focused on four main areas of resource allocation, namely, complete enclosure redesign, in situ welfare interventions, research project investment and boosting staff capacity (development of new positions). Across the three zoos, eight enclosures have either been fully renovated or are in the process of redesign. A further 195 small-scale interventions were actioned including lighting changes, feeding regime adjustment, the addition of more heating, cooling and/or shelter, increase in enclosure furniture for behavioural opportunities, the provision of sound proof retreats, increase in visual barriers, increased enrichment provision, diet reviews, additional visitor barriers, the provision of remote monitoring equipment, and increased keeper time spent in training for proactive health care across species. 

The process and its assessment outcomes have also proved useful for determining research priorities. Through the analysis of the ‘unknown’ scores across indicators/measures and across enclosures, gaps in understanding have been identified. This has led to the development of research projects aimed at addressing these gaps. As a result of the assessments and lack of information on the effects of certain environment conditions on animals, 14 research projects have been developed and completed or are presently underway across the three zoos. The majority of this research focuses on animal behaviour analyses to address the ‘unknowns’ in the behaviour domain; others are designed to investigate the impact of various conditions on the animals such as noise levels, lighting regimes, and visitor effects.

Lastly, investment in staff capacity building has resulted in several new positions being created across the three zoos including Animal Training Coordinators at each zoo and two new welfare research assistant positions. The data highlighting the ‘unknowns’ were used to demonstrate a business case to employ the research assistants to help conduct the applied projects described above.

Another indirect benefit of the use of this process has been the creation of an environment of open communication about animal welfare amongst zoo staff. The process enabled facilitated discussions to take place and provided opportunities for staff to raise welfare concerns in a systematic manner. As a result, this process is likely to have benefits for the quality of information gathered, but also benefits for staff education, awareness raising, and the fostering of a positive staff culture around animal welfare. This was not the primary aim of the process so no data were collected on whether or not demonstrable changes occurred, however managers at the three zoos involved anecdotally supported this notion.

### 4.3. Other Considerations and Limitations of Use

As highlighted above, the welfare assessment process provides zoos with the opportunity to conduct systematic risk assessments of animal welfare and develop evidence-based welfare work plans to prioritise welfare actions. This is an important part of a holistic animal welfare strategy for zoos, but it is important to note that this process should not be used as the sole means for evaluating animal welfare at an institute. Resource assessments can highlight the importance of certain ways of providing for the biological needs of a species, but it is also recognised that the provision of these factors alone does not necessarily translate to good welfare for the animal [29,32]. The inclusion of animal-based measures is therefore intended to present a qualitative, yet informative, assessment of how animals utilise the resources provided. However, this process is still predominantly resource-based (75% of the criteria), with a smaller proportion of animal-based measures (25%), and that is why it is referred to as a risk assessment process rather than a welfare assessment process. However as our knowledge improves, further animal-based measures can be incorporated into the process. Moreover, its use may be enhanced by supplementation with additional processes designed to provide more detail on individual animal welfare metrics, supported by applied research focused on behavioural and physiological changes in individuals. 

This trial set the welfare threshold for prioritisation relatively high (at 60%), however this should be assessed on a case by case basis for use at other institutions based on their distribution of data. It is also important for benchmarking that the threshold does not shift considerably between years to allow meaningful comparison to occur over time. However, as evidenced in the present dataset, the spread from minimum-to-maximum scores can and would be expected to change, and the goal should ultimately be to increase the benchmark level as progress is achieved in order to foster continuous improvement. In addition to prioritizing the enclosures falling below the set threshold, institutions may also wish to set a standard to improve all enclosures scoring in the bottom 5 or 10%, as this should foster further continuous improvement in welfare regardless of any potential biases in the rating involved in the use of the process. Moreover, it is important that, from the outset, such a process is recognised as adaptable. We will continue to learn more about species needs and preferences and it is critical that these scientific advances inform our consideration of the standards against which we will gauge success for each species. Zoos need to strive for goals well beyond the legislated minimum standards.

Other considerations for the use of this process relate to the personnel and resources required and the potential for inherent bias in the judgements made. Firstly, annual assessment using the process can be resource intensive for zoos because of the number of people and time involved (keepers, veterinarians, managers, and a welfare specialist); the time required will depend on the number of enclosures being assessed. Large zoos with hundreds of enclosures will require a larger portion of time for data collection, analysis, and reporting, meaning annual assessments are likely to be most feasible. However smaller zoos with fewer enclosures may be in a position to conduct more frequent assessments, which has the benefit of providing more data over time which may fasten progress. In addition, the success of the process relies on an effective chair to enable balanced discussion and ensure all participants have the opportunity to meaningfully contribute. Judgements about the level of risk or welfare are necessarily qualitative; as such, the accuracy of such judgements is likely to vary with experience and education/training among the team of experts. There may also be a risk of judgements being too negative or too positive according to the perceptions and motivations of the scorers. For example, if there is a sense that judgement is being made about the level of care offered by the keepers, there may be a risk of a positive bias in scoring. In contrast, if the process is seen to be an effective way to receive resources, there may be risk of a negative bias. Again, this relies on an effective chair and/or welfare specialist to manage.

At a more general level, the lack of sector-wide agreed ‘positive’ welfare standards for many species, as well as a lack of information for some species regarding natural behavioural biology, can make requirements difficult to judge for some species; this predisposes towards assignment of the ‘unknown’ option. Related to this, the major focus of the assessment is on risk identification, i.e., identification and minimization of negative experiences and negative welfare states. This may focus users towards achieving neutral welfare states, rather than striving for positive states for animals. Again, identifying appropriate targets for the assessments relies both on welfare-specific education of scorers and on an effective chair to ensure that sufficient focus is placed on positive welfare. 

## 5. Conclusions

This paper outlines the development and testing of a zoo-specific welfare risk assessment process. Whilst acknowledging the above limitations of its use, the process offers considerable value to the zoo sector in facilitating evidence-based management through highlighting areas of risk that need to be addressed, and by identifying opportunities to further improve welfare standards. Its use may also offer indirect benefits by fostering a staff culture of openness about animal welfare, educational benefits in animal welfare risk identification, a focus on continuous improvement in animal welfare management, and the integration of animal welfare as a whole of institution matter.

This welfare risk assessment process is an important part of a holistic animal welfare strategy for zoos, with its role positioned at an institutional level of assessment. Thus, it is important to ensure its use is supplemented by more detailed individual animal welfare monitoring processes for target species that focus on animal-based outcomes, as well as applied research dedicated to advance our understanding of species needs and preferences in zoos.

It represents a further step towards achieving high-level animal welfare in zoos by integrating animal welfare as an institutional priority alongside conservation. The more zoos that employ such strategies, the better will be the ability of the sector as a whole to advance the welfare of the animals in their care.

## Figures and Tables

**Table 1:** List of assessment indicators/animal-based measures, categorised across four domains of welfare. The remainder are considered resource-based risk factors.

**Environment (Physical/Social)**	**Focus of the Question**
1. There is adequate space for the animal(s) to perform required behaviours (including activity and retreat)	Space allowance
2. Enclosure design/features provide for a range of species-appropriate behaviours (e.g., flight, swimming, climbing, and digging)	Complexity: provision of species-appropriate behavioural opportunities in enclosure
3. Enclosure substrate is varied and appropriate (e.g., mix of soft/hard, provides for sensory stimulation, and good condition in all seasons)	Substrate quality and variation
4. Enclosure features adequately consider sensory environment relevant for species (e.g., visual barriers/retreats, long range views, sound buffers, perimeter and height of exposure to visitors)	Sensory environment: vision, sound, olfactory, and tactile
5. Enclosure keeps animals safe from potential threats (e.g., visitor incursion, wild predators, rodents, etc.)	Animal safety
6. Animals have access to varied and appropriate climate range that provides choices (e.g., shade, heating, wind and rain protection, ventilation, and lighting)	Access to appropriate thermal range
7. Individuals are housed in appropriate social/group settings for the species (e.g., solitary or social and ratio of males to females)	Social group
8. Environment and/or facilities on site support introduction of new animals and separation of individuals if required	Facilities to allow effective management of the individual or group
**Behaviour**	
9. Enrichment is species-specific, supports varied behavioural outcomes and is delivered according to program (e.g., tactile, olfactory, food-based, and cognitive)	Behavioural opportunities through enrichment
10. Behavioural time budgets for species/individual are considered optimal (focussing on time spent engaged in each activity, considering 24 h timeframe)	Time spent engaged in various behaviours
11. Presence of abnormal behaviour (excessive aggression and/or avoidance, stereotypies, etc.)	Presence or absence and frequency of abnormal behaviour
12. Species is known to display species-typical diversity in their behaviour, including positive behaviours (e.g., play, exploration, positive social interaction)	Range of behavioural repertoire observed
13. Food is presented in a way that encourages natural foraging behaviour	Food presentation for behavioural opportunities
**Physical Health/Nutrition**	
14. Varied, species appropriate diet and adequate supply of fresh drinking water of appropriate temperature is accessible for all individuals	Diet and drinking water
15. Animals appear to be in appropriate body condition for the individual’s age/sex/season (e.g., appropriate weight and coat/feather/skin condition)	Body condition (including weight and coat/feather/skin condition)
16. Proactive health care and parasite management is provided (e.g., supplements and parasite screening) and animals are free from injury and/or disease, or if present, these are being managed appropriately	Proactive health care; injury or disease
**Husbandry**	
17. Animals can be readily observed and adequate time is spent monitoring animals (e.g., visual check or remote monitoring and observation time allowance)	Time available to monitor, as well as physical ability to check on animals
18. Animals have a neutral or positive relationship with keepers- show no abnormal fear response to daily keeper interactions	Human-animal relationships
19. There is a flexible routine that allows the animal choice and considers natural activity patterns of the species—inclusive of visitor encounters	Flexibility, predictability of routines, and choice
20. Training program to facilitate proactive health care for the species requirements	Training for future health management

**Table 2:** Outline of the scoring process. ‘Unknown’ can be assigned for either resource-based risks or animal-based risks.

Score	Description
**Resource-Based Risk Level**	
0	High risk: E.g., resource considered to be inadequate for animal and likely to have welfare implications
1	Moderate risk: E.g., resource considered to be suboptimal and improvements needed
2	No observable risk: E.g., resource provision considered to be good and species-appropriate according to natural behavioural biology
**Animal-Based Welfare Level**	
0	Poor: E.g., animals either under or over weight; behavioural abnormality present; limited behavioural diversity observed compared to that expected for the species; shows little engagement with and is excessively fearful of keepers
1	Moderate: E.g., animals slightly over or under weight; have observed signs of behavioural abnormality but not frequent; displays limited behavioural repertoire; somewhat engaged with environment and keepers
2	Good: E.g., animals in good condition; no signs of behavioural abnormality; displays high levels of behavioural diversity as expected for the species; appears engaged in environment and with keepers
**Unknown**	Team considers they do not have information critical to making a judgement

**Table 3:** Total number of enclosures assessed, the mean welfare score, standard error, and median welfare risk at each zoo in each year of the trial.

Measure	2015	2016	2017
**Zoo 1**			
Number of enclosures assessed	101	93	107
Average welfare risk score	1.73	1.67	1.50
Standard error	0.02	0.03	0.03
Median welfare risk	1.80	1.72	1.5
**Zoo 2**			
Number of enclosures assessed	74	65	63
Average welfare risk score	1.82	1.80	1.45
Standard error	0.03	0.02	0.03
Median welfare risk	1.90	1.83	1.47
**Zoo 3**			
Number of enclosures assessed	45	44	36
Average welfare risk score	1.61	1.69	1.21
Standard error	0.04	0.03	0.05
Median welfare risk	1.70	1.72	1.24
